# Mechanical Characterization of Porous Bone-like Scaffolds with Complex Microstructures for Bone Regeneration

**DOI:** 10.3390/bioengineering12040416

**Published:** 2025-04-14

**Authors:** Brandon Coburn, Roozbeh Ross Salary

**Affiliations:** 1 Department of Mechanical & Industrial Engineering, Marshall University, Huntington, WV 25755, USA; 2 Department of Biomedical Engineering, Marshall University, Huntington, WV 25755, USA

**Keywords:** advanced manufacturing, bone tissue engineering, regenerative medicine, triply periodic minimal surface (TPMS)

## Abstract

The patient-specific treatment of bone fractures using porous osteoconductive scaffolds has faced significant clinical challenges due to insufficient mechanical strength and bioactivity. These properties are essential for osteogenesis, bone bridging, and bone regeneration. Therefore, it is crucial to develop and characterize biocompatible, biodegradable, and mechanically robust scaffolds for effective bone regeneration. The objective of this study is to systematically investigate the mechanical performance of SimuBone, a medical-grade biocompatible and biodegradable material, using 10 distinct triply periodic minimal surface (TPMS) designs with various internal structures. To assess the material’s tensile properties, tensile structures based on ASTM D638-14 (Design IV) were fabricated, while standard torsion structures were designed and fabricated to evaluate torsional properties. Additionally, this work examined the compressive properties of the 10 TPMS scaffold designs, parametrically designed in the Rhinoceros 3D environment and subsequently fabricated using fused deposition modeling (FDM) additive manufacturing. The FDM fabrication process utilized a microcapillary nozzle (heated to 240 °C) with a diameter of 400 µm and a print speed of 10 mm/s, depositing material on a heated surface maintained at 60 °C. It was observed that SimuBone had a shear modulus of 714.79 ± 11.97 MPa as well as an average yield strength of 44 ± 1.31 MPa. Scaffolds fabricated with horizontal material deposition exhibited the highest tensile modulus (5404.20 ± 192.30 MPa), making them ideal for load-bearing applications. Also, scaffolds with large voids required thicker walls to prevent collapse. The *P.W. Hybrid* scaffold design demonstrated high vertical stiffness but moderate horizontal stiffness, indicating anisotropic mechanical behavior. The *Neovius* scaffold design balanced mechanical stiffness and porosity, making it a promising candidate for bone tissue engineering. Overall, the outcomes of this study pave the way for the design and fabrication of scaffolds with optimal properties for the treatment of bone fractures.

## 1. Introduction

### 1.1. Background

Triply Periodic Minimal Surface (TPMS) scaffolds are a class of biomimetic structures characterized by their continuous, periodic surfaces with zero mean curvature. These mathematically derived geometries are increasingly used in bone tissue engineering due to their exceptional mechanical and biological properties. TPMS scaffolds offer a unique combination of strength, lightweight design, and permeability, making them ideal candidates for supporting bone regeneration. Their complex, interconnected porous networks mimic the trabecular architecture of natural bone, providing an optimal environment for cell attachment, proliferation, and nutrient exchange. Additionally, the large surface area-to-volume ratio of TPMS scaffolds enhances bioactivity by facilitating the adhesion of osteoblast cells and promoting the formation of new bone tissue.

From a mechanical perspective, TPMS scaffolds demonstrate excellent load-bearing capacity and energy absorption, closely resembling the mechanical behavior of cancellous bone. Their continuous surfaces distribute stress uniformly, reducing the risk of stress concentration and mechanical failure. By carefully tailoring design parameters such as unit cell type, pore size, strut thickness, and porosity, the mechanical properties of TPMS scaffolds can be optimized to meet specific clinical requirements. This tunability is particularly advantageous for patient-specific bone implants, where scaffold designs can be customized to match the mechanical properties of the surrounding bone, reducing the risk of implant failure or stress shielding.

In addition to their mechanical performance, TPMS scaffolds offer significant biological advantages. The interconnected porosity facilitates vascularization and tissue ingrowth, which are critical for effective bone regeneration. The porous architecture allows for the diffusion of nutrients, oxygen, and waste products, creating a conducive environment for cellular activity. Moreover, TPMS designs can be fabricated using bioresorbable materials such as calcium phosphate-based ceramics, biopolymers, or metallic alloys like titanium, which are biocompatible and promote osteointegration. The degradation rate of the scaffold can be controlled to match the rate of new bone formation, ensuring reliable replacement by natural bone tissue over time.

However, despite these advantages, the mechanical performance of TPMS scaffolds can vary significantly based on their design parameters. Variations in pore geometry, unit cell configuration, and material properties influence the scaffold’s ability to withstand compressive loads and maintain structural integrity. Additionally, while TPMS scaffolds can offer improved mechanical stability compared to traditional porous scaffolds, they may exhibit different failure modes under complex loading conditions. Therefore, a systematic investigation of scaffold design and mechanical characterization is necessary to ensure their suitability for bone tissue engineering applications.

Characterizing the mechanical properties of TPMS scaffolds involves assessing their stress-strain behavior under compressive, tensile, or shear loading. Evaluations of both the elastic and inelastic regions provide insights into the scaffold’s stiffness, yield strength, and post-yield behavior. Stress-strain analysis allows researchers to compare the mechanical performance of different scaffold designs, identify potential failure points, and optimize the design for enhanced performance. Additionally, experimental validation through mechanical testing, combined with computational simulations using, for example, finite element analysis (FEA), can further improve the predictive accuracy of scaffold behavior under physiological conditions.

### 1.2. Literature Review

The application of TPMS structures in bone regeneration has shown significant promise across various studies, highlighting the importance of pore size, interconnectivity, and pore geometry in influencing cellular behavior and mechanical performance.

Dong et al. examined TPMS scaffolds, which replicate trabecular bone-mimicking hyperboloidal topography to support cellular stem cell development. The study analyzed the impact of pore size, porosity, and pore shape parameters on key properties such as mechanical strength, permeability, and curvature, providing insights into their role in scaffold performance [1]. The high interconnectivity and hyperboloidal topography of TPMS scaffolds promote cellular processes essential for bone healing. In addition, they provide a conducive environment for cell attachment, proliferation, and differentiation, which are crucial for successful bone regeneration.

Naghavi et al. investigated the mechanical properties of compression, tension, bending, and torsion for load-bearing implants, focusing on gyroid and diamond TPMS designs. The study evaluated their porosity, stiffness, and strength to assess their suitability for bone replacement. Findings revealed that both TPMS designs exhibited stiffness and strength comparable to cortical bone and demonstrated bone mimicry in both physical and mechanical properties, confirming their viability for bone tissue engineering applications [2]. They reported that gyroid scaffolds with pore sizes ranging from 600 to 1200 µm exhibit mechanical properties suitable for load-bearing orthopedic implants. The high interconnectivity of these TPMS structures ensures efficient nutrient and waste exchange, which is vital for maintaining cell viability and function. The study also highlighted the importance of pore geometry, with gyroid and diamond structures providing optimal mechanical stability and support for bone tissue engineering.

Du et al. investigated additively fabricated polyether ether ketone/silicon nitride (PEEK/SiN) scaffolds with a TPMS architecture, highlighting their large surface area and uniform stress distribution advantages. The TPMS scaffolds exhibited favorable damping characteristics and mimicked trabecular bone’s physical properties. Additionally, the PEEK/SiN material promoted osteogenic differentiation, making it a promising candidate for bone tissue engineering applications [3]. They found that the TPMS-based PEEK/silicon nitride scaffolds, with 30% porosity, stimulate osteogenic differentiation, making them promising for spinal fusion applications. The interconnected porous network of these scaffolds mimics the natural bone architecture, facilitating the infiltration of osteogenic cells and promoting bone formation. The study underscores the significance of pore size and interconnectivity in enhancing the biological performance of TPMS scaffolds.

Khrapov et al. fabricated TPMS gyroid scaffolds using two different Electron Beam Melting (EBM) methods and evaluated their surface morphology, geometry, and mechanical properties using electron microscopy, X-ray imaging, and mechanical testing. The different fabrication methods resulted in variations in wall thickness, with the quasi-elastic method producing an elastic modulus of 1.5 GPa, closely resembling human bone. Finite Element analysis revealed that elastic wall regions were the primary sites of deformation. Both EBM methods successfully produced gyroid structures capable of mimicking bone’s mechanical properties, making them viable for biomedical applications [4]. Particularly, it was observed that gyroid sheet-based structures with a wall thickness of 200 µm, fabricated using electron beam melting, exhibit mechanical properties similar to human cortical bone. The high interconnectivity and precise pore geometry of these scaffolds contribute to their mechanical strength and biocompatibility, making them suitable for orthopedic applications. This study highlights the potential of TPMS structures in replicating the mechanical behavior of natural bone.

Mishra et al. explored the impact of material variation on the uniaxial compression behavior of FDM-manufactured polymeric TPMS lattice materials, specifically comparing PLA and ABS [5]. Focusing on Schwarz Primitive (P) lattices, the mechanical behavior of these materials was examined under varying strain rates, ranging from 5 mm/min to 35 mm/min. The results indicated that PLA samples exhibited approximately 10% lower compressibility than ABS samples. Furthermore, the first peak stress increased linearly with strain rate, with PLA samples exhibiting up to 30% higher peak stress values compared to ABS. Overall, this study highlights the importance of material selection in determining the mechanical performance of TPMS structures, which is crucial for their application in biomedical implants and bone tissue engineering.

Tilton et al. investigated the environmental dependency of mechanical responses in additively manufactured porous polymethyl methacrylate (PMMA) scaffolds, utilizing Primitive and Schoen-IWP designs. The scaffolds were tested under compressive loading in both ambient and fluidic environments (with pH = 7.4), revealing a significant reduction in compressive performance under fluidic conditions. Despite this loss, the compressive properties and flexural stiffness remained within the range of trabecular bone for both scaffold designs. This can be attributed to the high interconnectivity as well as the specific pore geometry of the designs. The study further demonstrated that the mechanical behavior of additively manufactured scaffolds is primarily governed by topology and morphology, highlighting the importance of structural design in optimizing scaffold performance for biomedical applications [6].

De Aquino et al. investigated the mechanical effects of fabrication orientation on TPMS scaffolds produced via FDM. The study analyzed the compressive response of scaffolds fabricated at 0° and 90° orientations, revealing significant variations in compressive modulus based on directional effects. Three TPMS designs, i.e., Primitive, Gyroid, and Diamond, were evaluated, with the Primitive design demonstrating the best compressive performance. The findings highlight that loading direction plays a crucial role in determining the compressive strength and elastic modulus of TPMS scaffolds, emphasizing the importance of fabrication orientation in scaffold design for biomedical applications [7]. In addition, the high interconnectivity and precise pore geometry of these scaffolds contribute to their compressive strength and overall mechanical performance.

Hameed et al. studied the mechanical properties of Gyroid TPMS scaffolds with varying pore sizes, fabricated using selective laser melting (SLM). They found that larger pore sizes resulted in lower compressive strength, with the 250 µm pore size achieving a compressive strength of 205 MPa. Additionally, biocompatibility tests using human mesenchymal stem cells (hMSCs) indicated that scaffolds with larger pore sizes had the highest biocompatibility [8]. Overall, this study underscores the significance of pore size in enhancing the mechanical and biological performance of TPMS scaffolds. The high interconnectivity and specific pore geometry of Gyroid structures contribute to their suitability for bone implant applications.

Maevskaia et al. examined the mechanical performance and biological effects of 3D-cell culture on Diamond, Gyroid, and Primitive scaffolds made of hydroxyapatite, all with a minimal constriction of 0.8 mm diameter. They found that Gyroid and Diamond scaffolds had significantly higher compression strength compared to Primitive (P) and lattice designs. In-vitro culture of human bone marrow stromal cells showed no differences among the scaffolds. However, in-vivo tests revealed that Diamond and Gyroid scaffolds had higher bone ingrowth and bone-to-implant contact, making them the most effective for bone tissue regeneration [9]. These findings highlight the importance of pore geometry and interconnectivity in optimizing the mechanical and biological performance of TPMS scaffolds for bone tissue engineering.

Castro et al. studied the mechanical properties of a TPMS Gyroid design with 50% and 70% porosity, using 3D MultiJet printing for accurate fabrication, highlighting the importance of elasticity for cellular growth and proliferation [10]. The high interconnectivity of the fabricated Gyroid structures ensures efficient nutrient and waste exchange, which is crucial for maintaining cell viability and function. The Gyroid geometry provides a balance between mechanical strength and biological performance. Scaffold stiffness is a function of porosity, with higher porosity leading to lower stiffness. This relationship is essential for designing scaffolds that can support cellular behavior while providing adequate mechanical support.

Valainis et al. explored the fabrication of TPMS scaffolds (based on the Diamond, Gyroid, and Schwarz Primitive designs) using polycaprolactone (PCL). They also conducted a finite element model to evaluate Young’s modulus and performed in-vitro studies to assess cell migration. The results showed that adipose-derived mesenchymal stromal cells (AdMSC) migrated farther on the scaffolds, with migration rates increasing when the scaffolds were coated with calcium-phosphate-based apatite [11]. In addition, the finite element analysis demonstrated that the Young’s modulus (stiffness) of the scaffolds can be effectively tailored to mimic that of natural bone by fine-tuning the design parameters. Overall, it is implied from this study that optimized pore geometry and enhanced interconnectivity can significantly promote cell attachment, proliferation, and differentiation.

Cai et al. studied the impact of porosity on the mechanical properties of periodic cellular structures using various TPMS scaffold designs with 55% and 80% porosities (including Skeletal-IWP, Skeletal-Diamond, Skeletal-Gyroid, Sheet-Gyroid, Sheet-Primitive, Sheet-IWP, and Sheet-Diamond). They conducted compressive analyses and found that low porosity variants had yield strengths 3 times higher as well as stiffness 2.5 times larger than high porosity variants. It addition, it was observed that high porosity structures failed via buckling, while low porosity ones failed via micro-fracturing [12]. Overall, this study underscores the vital relationship between porosity and mechanical behavior in the design of scaffolds, emphasizing the need to balance structural integrity with the support of cellular activity. By optimizing porosity, scaffolds can effectively promote cellular behavior while ensuring sufficient mechanical support for bone tissue engineering applications.

Yang et al. studied the fatigue behavior and underlying failure mechanisms of Gyroid structures [13]. Gyroid structures exhibit smooth surface connections between struts, which contribute to their superior fatigue resistance properties. The study revealed that cyclic ratcheting as well as fatigue damage are key contributors to the failure of these Gyroid structures, with fractured samples exhibiting distinct 45° fracture bands along the diagonal surface. Additionally, sandblasting treatment was shown to enhance fatigue resistance by effectively removing adhered powder particles. The high interconnectivity and precisely controlled pore geometry of Gyroid structures promote cell penetration, vascular ingrowth, nutrient diffusion, and waste elimination, making them highly promising for long-term applications in dynamic bio-skeletal environments.

Gabrieli et al. explored the use of additive manufacturing and computational methods to create porous scaffolds with complex microstructures and mechanical performance similar to cancellous bone. They focused on TPMS scaffolds with Schwarz Primitive and Schwarz Gyroid designs, which feature highly interconnected pore geometries. The relationship between relative density and elastic modulus was analyzed, showing that larger wall thickness reduces elastic modulus as well as compressive strength. This finding underscores the importance of optimizing pore geometry and interconnectivity to enhance the mechanical performance of scaffolds [14].

Vijayavenkataraman et al. studied 12 different versions of porous bone implant designs based on the Schwarz Primitive design, fabricated from alumina using Lithography-based Ceramics Manufacturing (LCM) technology. These designs had pore sizes ranging from 500 to 1000 μm and porosity of above 50%. The intricate architecture of these TPMS structures presents numerous advantages, including reduced stress concentration, excellent permeability, and a high surface area-to-volume ratio. These features create an optimal environment for cell adhesion, migration, and proliferation. The study revealed that the material composition and porosity of the Schwarz Primitive design yielded a compressive modulus comparable to that of native bone. This characteristic not only has the potential to mitigate stress-shielding effects but also promotes favorable cellular behavior, making it a promising candidate for bone tissue engineering applications. [15].

### 1.3. Gaps and Objectives

The previous section provided a review of the literature, highlighting the mechanical and biological properties as well as applications of TPMS bone scaffolds, which have demonstrated potential in bone tissue engineering due to their high surface area, interconnected porosity, and biomimetic architectures. These scaffolds play a crucial role in supporting cell adhesion, proliferation, and differentiation, which are essential for osteogenesis and bone regeneration. However, despite research on some TPMS designs, a significant knowledge gap remains in the relative understanding of the functional performance of a broader range of TPMS structures, particularly in relation to their internal microstructures. Therefore, the objective of this study is to address this gap by conducting an investigation of the mechanical properties of 10 different TPMS scaffold designs fabricated using fused deposition modeling (FDM) additive manufacturing. Specifically, this research work will evaluate their compressive stiffness and structural stability and will identify optimal TPMS structures that can enhance mechanical support for bone repair and regeneration.

Furthermore, while conventional biomaterials such as polyether ether ketone (PEEK), polycaprolactone (PCL), and polylactic acid (PLA) have been extensively studied and used in scaffold fabrication, the performance and viability of novel composite materials such as SimuBone remain largely unexplored. SimuBone, a biocompatible and biodegradable material, holds the potential for bone regeneration due to its mechanical resilience and osteoconductive properties. Therefore, the second objective of this study is to evaluate SimuBone’s mechanical properties (along with the assessment of the 10 TPMS designs), paving the way for establishing it as a candidate material for next-generation bone implants.

By addressing these gaps, this research aims to enhance the understanding of TPMS scaffold architectures as well as their mechanical behavior in bone tissue engineering. The insights gained from this study will contribute to the development of more effective, durable, and patient-specific bone scaffolds, ultimately advancing the field of regenerative medicine. In this study, the material and methods, including the design and fabrication of the TPMS scaffold designs, are described in Section 2. The results, including torsion, tensile, and compression analyses, are presented and discussed in Section 3. Finally, the conclusions and future work are presented in Section 4.

## 2. Materials and Methods

### 2.1. Materials

A medical-grade, biodegradable, ISO-certified, and antibacterial composite material, SimuBone (3DXTECH, Grand Rapids, MI, USA) was used for the fabrication of bone-like bone scaffolds. This advanced material not only meets stringent medical safety standards but is also designed to degrade safely within the body, reducing the need for additional surgical interventions to remove the scaffolds. The inclusion of antibacterial properties further enhances the material’s suitability for bone regeneration, as it helps prevent post-surgical infections, thereby promoting a safer healing environment. Additionally, the radio opaque nature of SimuBone allows for enhanced visibility on X-ray and CT scans, which is crucial for monitoring the scaffolds’ integration with the surrounding bone tissue and ensuring proper alignment during and after implantation (which will be part of the authors’ future studies). This visibility aids clinicians in making accurate assessments and timely decisions throughout patients’ recovery process, ultimately contributing to more successful surgical outcomes.

### 2.2. Methods

#### 2.2.1. Scaffold Design

**Figure 1:**
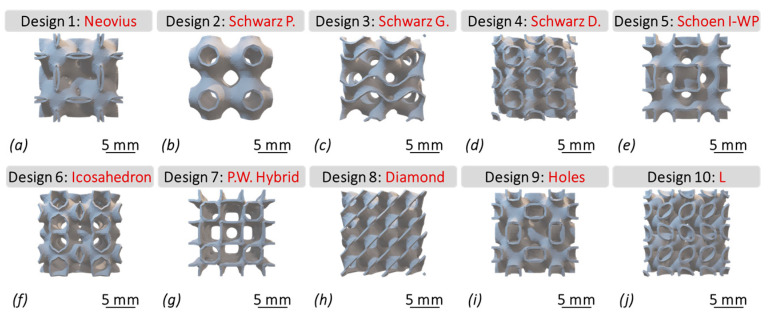
The morphology of 10 TPMS scaffold designs with complex porous microstructures, including: (**a**) Desing 1: Neovius, (**b**) Desing 2: Schwarz Primitive (P), (**c**) Desing 3: Schwarz Gyroid (G), (**d**) Desing 4: Schwarz Diamond (D), (**e**) Desing 5: Schoen I-WP, (**f**) Desing 6: Icosahedron, (**g**) Desing 7: P.W. Hybrid, (**h**) Desing 8: Diamond, (**i**) Desing 9: Holes, and (**j**) Desing 10: L.

As depicted in Figure 1, porous bone scaffolds were parametrically designed based on the fundamental principles of triply periodic minimal surfaces (TPMS). These surfaces are characterized by minimal areas and boundaries defined by closed curves, allowing for the creation of structures that efficiently balance strength and porosity. Each unit cell within the scaffold designs is assigned a specific surface thickness, which is then integrated into a cubically symmetric scaffold that retains interconnected pores through periodic repetition. This design approach results in scaffold architectures that closely resemble the intricate microstructure of trabecular bone.

The porous nature of the TPMS scaffolds is particularly advantageous for bone tissue engineering, as it facilitates essential cellular processes. The interconnected pores enhance bone tissue integration by promoting oxygen diffusion, ion exchange, and nutrient transport, which are critical for cell survival and proliferation. Moreover, the biomimetic structure of the scaffolds supports the natural remodeling processes of bone, which will aid in the regeneration of healthy bone tissues [1].

It is implied from Figure 1 that Neovius (a), Schwarz P. (b), Schoen I-WP (e), Icosahedron (f), P.W. Hybrid (g), and Holes (i) all show to have simplistic yet deeply interconnected curvatures, compared to Schwarz G. (c), Schwarz D. (d), and Diamond (h) which have more complex curvatures and thus sound cubic structures. Additionally, design #10, i.e., L (j), shows a rich mixture of the two categories.

#### 2.2.2. Parametric Modeling

The TPMS scaffolds were parametrically designed using Rhino 7 (Rhinoceros, Seattle, WA, USA), with the Grasshopper extension (Version: 1.0.0007, 2024) serving as the primary tool for algorithmic modeling. Table 1 presents the governing mathematical equations of the 10 TPMS scaffold designs [16,17], corresponding to the order illustrated in Figure 1. To further enhance the design process, Millipede (Sawapan Design; Final Version) and Weaverbird (Version 0.9.0.1) functions were employed, enabling the transformation of these equations into 3D solid models. These advanced functions not only facilitated the visualization of the geometries but also allowed for precise control over the main structural parameters (listed in Table 2), ensuring the accuracy and functionality of the designs for scaffold fabrication.

As shown in Table 2, the *model dimensions* of the TPMS scaffolds were uniform, 6 × 6 mm in length. The *iteration step size* controls the surface generation and refinement of the scaffold geometries. It was set to 7.710 for designs (b) through (i), while designs (a) and (j) had an *iteration step size* of 18.710; this was done to enhance the topology of the scaffolds for the additive fabrication process (discussed in Section 2.2.3). Aiding in generation of a uniform geometry based on a given threshold, the *Merged Toggle* parameter is based on Boolean functions (True/False), which was set to “True” in this study. The *IsoValue* parameter controls pore size and, thus, the porosity of the scaffold geometries (using double-precision floating-point values), set to −0.269. The *ArrBox Count* function was used to construct multiple units, which were subsequently attached to create single solid structures. This parameter was set to 2, meaning a total of 4-unit cells were created, attached in the form of single solid structures, as shown in Figure 1. The *level* parameter was set to 1, affecting the number of subdivisions of each face on the scaffolds. Set to 0.15 mm, the *WBThickness* parameter directly controls the wall thickness of the generated scaffold designs while indirectly influencing their porosity.

#### 2.2.3. Scaffold Fabrication

**Figure 2:**
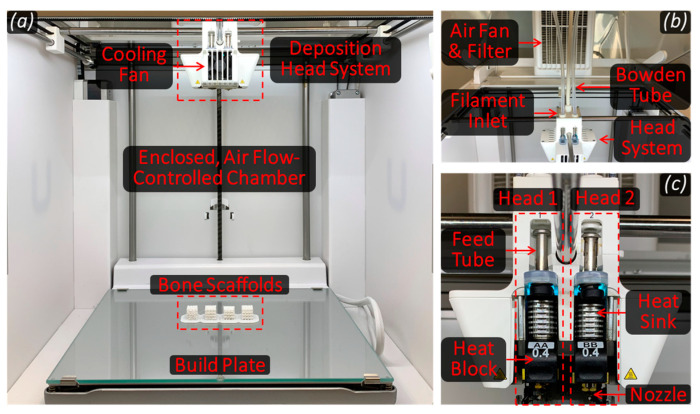
(**a**) The enclosed, air flow-controlled chamber of the fused deposition modeling (FDM) system used in this study showing the deposition head assembly as well as the build plate; (**b**) the upper section of the FDM system including an air fan, a filter, and Bowden tubes guiding filaments through the extrusion process; (**c**) the deposition head assembly components including two feed tubes, heat sinks, heat blocks, nozzles, as well as a cooling fan (visible when the assembly is closed).

**Figure 3:**
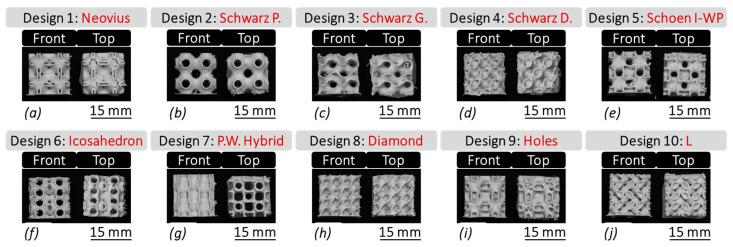
The front view as well as the top view of the FDM-fabricated TPMS bone scaffolds, including: (**a**) Desing 1: Neovius, (**b**) Desing 2: Schwarz Primitive (P), (**c**) Desing 3: Schwarz Gyroid (G), (**d**) Desing 4: Schwarz Diamond (D), (**e**) Desing 5: Schoen I-WP, (**f**) Desing 6: Icosahedron, (**g**) Desing 7: P.W. Hybrid, (**h**) Desing 8: Diamond, (**i**) Desing 9: Holes, and (**j**) Desing 10: L.

Porous TPMS-based bone scaffolds were fabricated using the fused deposition modeling (FDM) process. Figure 2 illustrates the main components of the FDM system, Ultimaker S5 (Utrecht, The Netherlands) used in this study. The medical-grade filament material, with a diameter of 2.85 mm, was fed into the system, where a stepper motor led the filament through a Bowden tube toward a heating block prior to deposition. The heating block controls the temperature and, thus, the rheological properties of the molten material. The molten polymer (flowing as a non-Newtonian fluid) was subsequently deposited on a heated surface using a microcapillary nozzle (400 µm in diameter). The temperature differential between the nozzle and the print bed surface (which was maintained at a fixed level with the aid of the chamber fan) facilitated rapid solidification and adhesion of the material to the surface, ensuring the stability and integrity of the printed scaffold structures. This deposition process was automatically repeated in accordance with a geometric code (g-code) generated using Cura (UltiMaker, Utrecht, The Netherlands, Version: 4.13.2 Enterprise), enabling layer-by-layer construction of the scaffolds. Each successive layer of molten polymer bonded to the previous one, resulting in the formation of robust and structurally sound bone scaffolds, as demonstrated in Figure 3.

The material deposition parameters used for the fabrication of all the 10 TPMS scaffold designs, torsion bar, and tensile bar are listed in Table 3. Please note that layer height refers to the thickness of each individual layer of material deposited, while layer width is the width of the individual lines of material extruded. In addition, infill density refers to the amount of material that has been deposited inside the scaffolds as a percentage of the total volume. In this study, the layer height used for the FDM fabrication of the bone scaffolds was 200 µm with a layer width of 300 µm. In addition, the infill density of the scaffolds and the test bars was set to 100%. The material deposition process was based on a microcapillary nozzle having a diameter of 400 µm. The bed temperature and the deposition head temperature were set to 60 °C and 240 °C, respectively. The printing speed of the FDM process was 10 mm/s, along with a flow rate of 120%. Furthermore, “brim” was the adhesion type used for all the 10 TPMS scaffolds as well as the test bars.

Please note that print speed significantly impacts material performance, influencing layer adhesion, surface quality, and structural integrity. A higher print speed can reduce printing time but may lead to poor interlayer bonding, increased porosity, and defects due to insufficient material deposition and cooling inconsistencies. Conversely, a lower print speed enhances adhesion and print accuracy but may cause overheating and material degradation. Optimal print speed depends on polymer viscosity, extrusion rate, and part geometry. Balancing speed with temperature and cooling settings ensures uniform deposition, minimizing warping and improving mechanical properties for high-quality, reliable material deposition.

Shown in Equation (1), *pore fraction* [%] was calculated based on a fabricated solid cube made up of the same scaffold material. The difference between the solid cube’s mass and that of the TPMS bone scaffolds was calculated and divided by the mass of the solid cube, resulting in the scaffold pore fraction, which is a measure of the pore/void fraction of the fabricated scaffolds.

(1) $$Pore\;Fraction\;\left[\%\right]=\left(\frac{Solid\;Cube\;mass-Scaffold\;mass}{Solid\;Cube\;mass}\right)\times 100\%$$

#### 2.2.4. Mechanical Characterization of Bone Scaffolds

**Figure 4:**
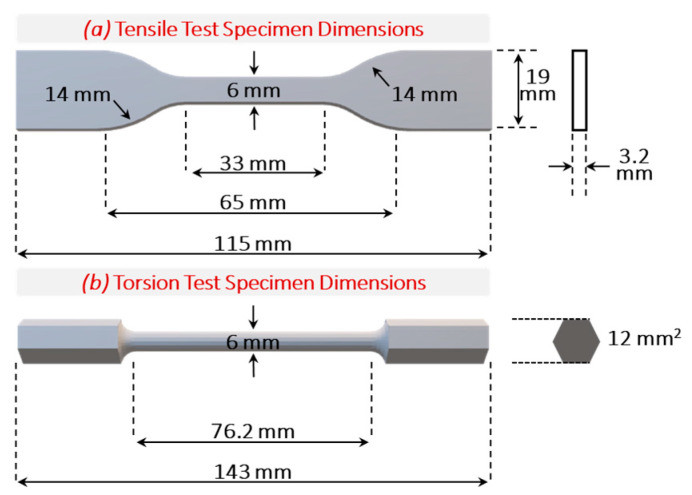
The dimensions of the specimens used for (**a**) the tensile test and (**b**) the torsion test, shown for each section using the dashed lines.

As demonstrated in Figure 4a, tensile specimens were fabricated in the form of a dog-bone, following the ASTM D638-14 (Type IV) standard design [18], with five specimens (*n* = 5) fabricated at three raster angles of 0°, 45°, and 90°. The Type IV design features a narrow section width of 6 mm and a narrow section length of 33 mm, optimized for accurate tensile analysis. The overall dimensions include a width of 19 mm and a length of 115 mm, providing a standardized format for consistent testing. The gage length, crucial for precise elongation measurement, is set at 25 mm, while the distance between the grips is 65 mm, ensuring adequate space for secure clamping during testing. The design also incorporates a radius of 14 mm on the fillets, contributing to the smooth transition between the narrow and wider sections and reducing stress concentrations. Additionally, the outer radius of 25 mm further supports the structural integrity of the specimen during testing, ensuring reliable and reproducible results.

A compression analysis was conducted on the fabricated TPMS bone scaffolds (as shown in Figure 3) using a precision compression testing machine (MTI-10K, Measurements Technology Inc., Marietta, GA, USA). The results of this analysis are instrumental in determining the scaffolds’ suitability for clinical applications, where mechanical stiffness and stability are paramount.

In addition, torsion specimens were fabricated, following the SM 1001 standard model illustrated in Figure 4b, with five specimens produced for testing (*n* = 5) [19]. The specimens were characterized using a torsion testing machine (TecQuipment, Model: SM1001, 30 Nm torque capacity, Nottingham, UK). Each torsion bar features a narrow section with a diameter of 6 mm and a length of 76.2 mm, designed to accurately assess the material’s torsional strength and behavior under twisting forces. The overall length of the torsion bar extends to 143 mm, ensuring sufficient leverage for the applied torsional loads. The outer sections of the torsion bar are designed with a cross-sectional area of 12 mm^2^, providing robust end sections for secure clamping during testing. Table 4 covers the parameters of the tensile and torsion designs used in this study for the characterization of the fabricated bone scaffolds.

## 3. Results and Discussion

The Results and Discussion section will integrate the findings from the torsion analysis (discussed in Section 3.2), tensile testing (outlined in Section 3.2), and compression performance (presented in Section 3.3). Additionally, this section will provide a discussion of the optimal performance of the TPMS scaffold designs, drawing comparisons between the mechanical responses observed in each type of loading scenario. By analyzing these results collectively, the interplay between the mechanical properties and the geometric features of the scaffolds will be evaluated to highlight the best-performing TPMS configurations across different mechanical tests.

### 3.1. Tensile Analysis

**Figure 5:**
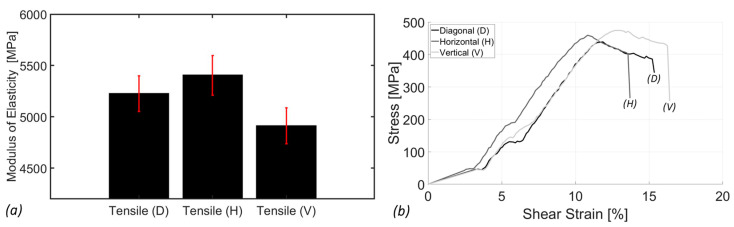
(**a**) The tensile modulus of elasticity (stiffness) and (**b**) a stress-strain plot of the bone scaffold biomaterial as a function of raster angle: (D): Diagonal (45°), (H) Horizontal (0°), and (V) Vertical (90°). The error bars (shown in red) represent the standard error of the sample data.

The tensile analysis provides insights into the mechanical properties of SimuBone under uniaxial tensile loading. Figure 5 illustrates the tensile modulus of elasticity of SimuBone as influenced by three raster orientations: 45° diagonal (D), 0° horizontal (H), and 90° vertical (V). Among these, the vertical orientation (90°) exhibited the lowest tensile modulus of elasticity (stiffness) at 4911.84 ± 175.18 MPa, while the horizontal orientation (0°) demonstrated the highest tensile modulus at 5404.20 ± 192.30 MPa, followed closely by the diagonal orientation (45°) at 5224.42 ± 173.77 MPa. These results indicate that the horizontal orientation (0°) is the preferred material deposition direction, as it provides the highest stiffness and structural integrity. Consequently, this orientation has been adopted for the development of torsion bars, as discussed in Section 3.2. Overall, SimuBone exhibits satisfactory tensile stiffness, comparable to that of cortical bone (14–20 GPa) [20] and cancellous (trabecular) bone (0.01–3 GPa) [21,22] (depending on factors such as bone density, anatomical location, and individual structural characteristics). Additionally, its low standard deviation across measurements highlights its consistency and reliability, making it a promising candidate for bone tissue engineering applications.

### 3.2. Torsion Analysis

**Figure 6:**
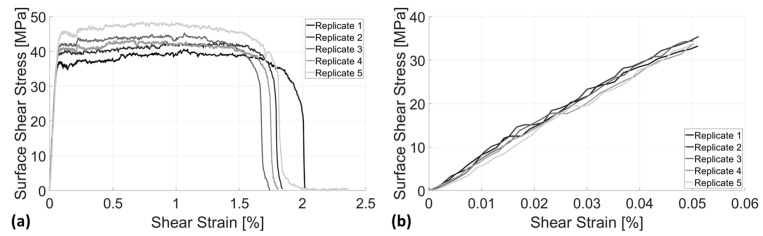
(**a**) A complete torsional stress-strain plot for the scaffold biomaterial utilized in this study, shown for both elastic and inelastic/plastic regions; (**b**) a torsional stress-strain plot shown only for the elastic region, for five replicates (*n* = 5).

The torsion analysis provides insight into the material’s mechanical properties under angular displacement stress. Figure 6a illustrates the total surface shear stress versus shear strain, demonstrating that Simubone exhibits a shear modulus of rigidity of 714.79 ± 11.97 MPa. This value was computed using an in-house MATLAB (Version: R2024a) code based on five replications to ensure accuracy and consistency. Additionally, the average ultimate yield strength across all five replications was determined to be 44 ± 1.31 MPa. As observed on the shear strain axis, necking and fracture occurred at approximately 1.5% and 1.75% strain, respectively, highlighting the material’s deformation characteristics. Furthermore, Figure 6b confirms the high consistency of the five replications, particularly in the linear region, validating the accuracy, repeatability, and reliability of the torsion test results.

### 3.3. Compression Analysis

**Figure 7:**
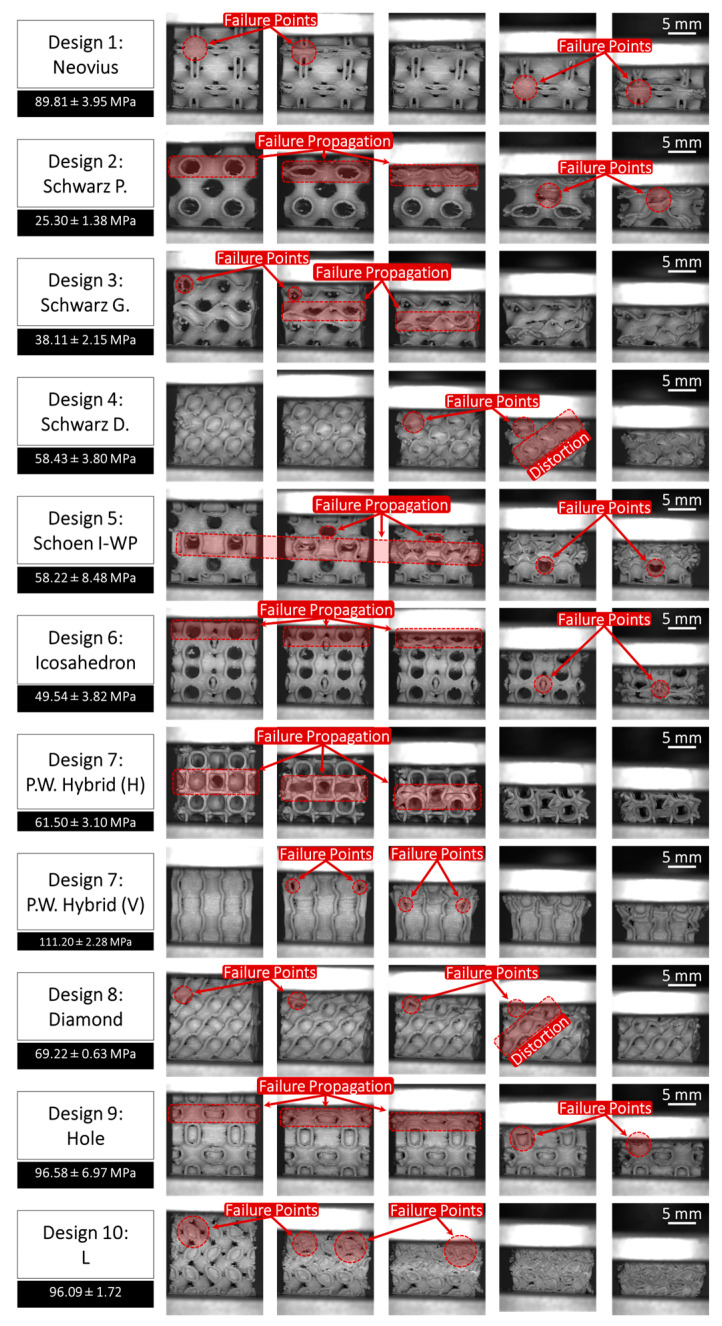
Visual demonstration of the compression behavior of the fabricated TPMS bone scaffolds (captured in real-time), including: (1) Neovius, (2) Schwarz Primitive (P), (3) Schwarz Gyroid (G), (4) Schwarz Diamond (D), (5) Schoen I-WP, (6) Icosahedron, (7) P.W. Hybrid, (8) Diamond, (9) Holes, and (10) L. Note that the P.W. Hybrid design was tested twice: horizontally (H) and vertically (V) because of the difference in the non-uniform alignment of the pores within the design.

**Figure 8:**
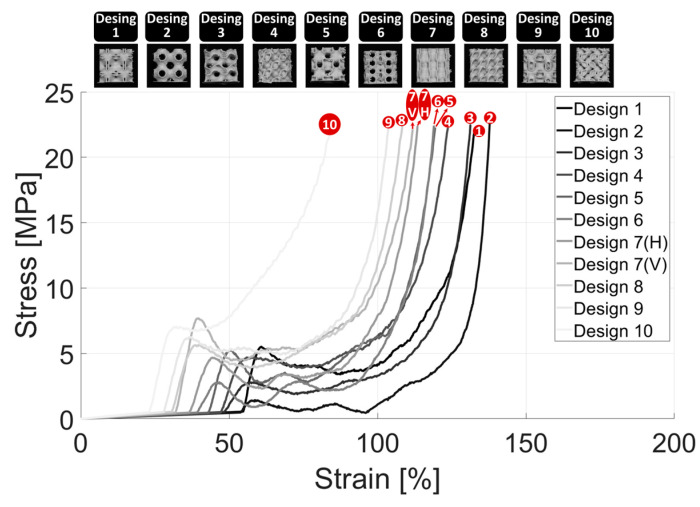
A stress-strain plot encompassing all characterized scaffold designs, illustrating both elastic and inelastic regions for a comparison of their mechanical behavior. The red colored numbers correspond to the 10 scaffold designs, as follows: Desing 1: Neovius, Desing 2: Schwarz Primitive (P), Desing 3: Schwarz Gyroid (G), Desing 4: Schwarz Diamond (D), Desing 5: Schoen I-WP, Desing 6: Icosahedron, Desing 7: P.W. Hybrid, Desing 8: Diamond, Desing 9: Holes, and Desing 10: L.

The scaffold compression analysis was conducted to systematically evaluate the impact of scaffold design (in terms of TPMS pore structure) on the compressive properties, pore percentage, and material deposition mass of the fabricated bone scaffolds. This analysis aimed to provide insights into how variations in the TPMS scaffold structures, and thus pore geometry, influence the overall structural integrity and mechanical performance of the scaffolds under compressive loading. Figure 7 illustrates the compression performance of all 10 fabricated TPMS scaffolds under progressive compressive loading (from left to right), providing critical insights into their failure mechanisms and structural integrity. In addition, Figure 8 provides a quantitative comparison of the mechanical performance of all 10 scaffold designs under compressive loads in both elastic and inelastic regions. Please note that the slope of the linear (elastic) region represents the stiffness of each scaffold. In addition, the deformation profile of the scaffolds in the inelastic/plastic region is a direct function of their internal structures. Scaffolds with relatively strong and packed internal structures, such as Design 4 (Schwarz Damond), experience less structural deformation and decline in the inelastic region in contrast to the scaffolds having large pores and thus relatively fragile internal structures, such as Design 2 (Schwarz Primitive).

Additionally, Figure 7 highlights the failure point locations within each scaffold architecture, revealing distinct deformation patterns under load. A common trend observed in the Schwarz Primitive (P), Schwarz Gyroid (G), Schoen I-WP, Icosahedron, and P.W. Hybrid (H) designs is the fracture occurring primarily within the void regions of the scaffolds, exhibiting pronounced architectural failure within their hollow regions, where the structures collapse inward upon itself. This suggests a need for enhanced wall thickness in these areas to improve mechanical stability and load-bearing capacity.

**Figure 9:**
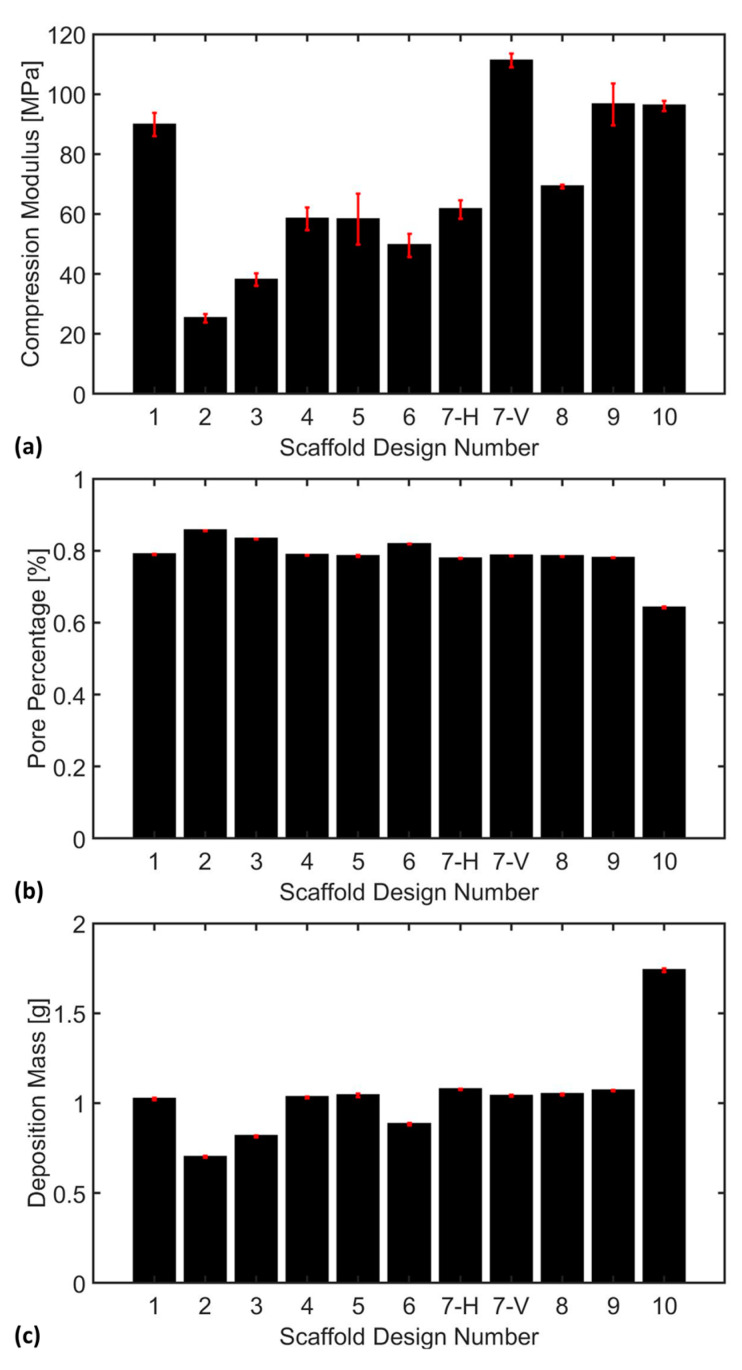
A comparison of (**a**) the compression modulus of elasticity, (**b**) pore percentage, and (**c**) deposition mass of the fabricated TPMS bone scaffolds. Note that the P.W. Hybrid design (i.e., #7) was tested twice: horizontally (H) and vertically (V). The error bars (in red) represent the standard error of the sample data. The 10 scaffold designs are: Desing 1: Neovius, Desing 2: Schwarz Primitive (P), Desing 3: Schwarz Gyroid (G), Desing 4: Schwarz Diamond (D), Desing 5: Schoen I-WP, Desing 6: Icosahedron, Desing 7: P.W. Hybrid, Desing 8: Diamond, Desing 9: Holes, and Desing 10: L.

Furthermore, the underlying support structures in several designs contribute to resistance, delaying failure, and enhancing overall mechanical performance. The progressive collapse of hollow voids, particularly in scaffolds with simple curvature and cubic structures, can be mitigated through strategic reinforcement in critical regions, such as incorporating thicker strut elements and decreasing pore opening. The scaffolds with enriched curvature and reduced pore openings, such as the Schwarz Diamond (D) design, exhibited a consistent failure pattern. The enhanced curvature of these scaffolds contributed to a more uniform stress distribution, delaying the onset of failure compared to the designs with thinner curvature and more open voids. Figure 9a provides a comparison of the compression moduli of all 10 TPMS designs.

As implied in Figure 9a, the weakest TPMS scaffold design is Scaffold #2, i.e., Schwarz Primitive (P), characterized by large voids and having a compression modulus of 25.30 ± 1.38 MPa. The Schwarz Primitive (P) is followed by Scaffolds #3 and #6, i.e., Schwarz Gyroid (G) and Icosahedron, respectively, having a stiffness of 38.11 ± 2.15 MPa and 49.54 ± 3.82 MPa. In addition, Scaffolds #4, #5, 7(H), and #8, i.e., Schwarz Diamond (D), Schoen I-WP, P.W. Hybrid (Horizontal), and Diamond, respectively, had a medium level of stiffness, namely 58.43 ± 3.80 MPa, 58.22 ± 8.48 MPa, 61.50 ± 3.10 MPa, and 69.22 ± 0.63 MPa. Finally, Scaffolds #1, #7(V), #9, and #10, i.e., Neovius, P.W. Hybrid (Vertical), Holes, and L, had a relatively high level of elasticity modulus, namely 89.81 ± 3.95 MPa, 111.20 ± 2.28 MPa, 96.58 ± 6.97 MPa, and 96.09 ± 1.72 MPa (attributed to their relatively compact and thicker internal structures).

Please note that Scaffold #7, i.e., P.W. Hybrid, was compressed vertically and horizontally due to its structure not being identical when rotated. It was observed that the compression modulus of the vertical (V) placement was far superior compared to that of the horizontal (H) placement. This is because, in the vertical (V) case, the scaffold collapses on top of its supportive regions, while in the horizontal (H) case, the scaffold lies on top of its pores, as implied in Figure 7.

It is observed in Figure 9b that almost all scaffold designs except Scaffold #2, #3, #6, and 10, i.e., Schwarz Primitive (P), Schwarz Gyroid (G), Icosahedron, and L, have a comparable pore percentage of approximately 78%. Scaffold #2, i.e., Schwarz Primitive (P), has the highest level of pore percentage (0.86 ± 0.0009%), followed by Scaffold #3 and #6, i.e., Schwarz Gyroid (G) and Icosahedron, having a pore percentage of 0.83 ± 0.0008% and 0.82 ± 0.0012%, respectively, while Scaffold #10, i.e., L, has the lowest amount of pore percentage (0.64 ± 0.0019).

Similarly, it is observed in Figure 9c that almost all scaffold designs except Scaffold #2, #3, #6, and #10, i.e., Schwarz Primitive (P), Schwarz Gyroid (G), Icosahedron, and L, have a comparable deposition mass of approximately 1 g. Scaffold #2, i.e., Schwarz Primitive (P), had the lowest amount of deposition mass (0.70 ± 0.004 g), followed by Scaffold #3 and #6, i.e., Schwarz Gyroid (G) and Icosahedron, while Scaffold #10, i.e., L, had the highest level of deposition mass (1.73 ± 0.009 g). It turned out that there was an inverse non-linear relationship between the pore percentage and deposition mass. However, no clear relationship was observed between the deposition mass and compression modulus, which may stem from the influence of internal pore structure on scaffold stiffness.

Overall, after evaluating all critical factors, Design #7 (P.W. Hybrid) exhibited a high level of stiffness in the vertical orientation but only a moderate stiffness in the horizontal direction. This pronounced anisotropic behavior suggests that P.W. Hybrid may not be the optimal choice for bone tissue engineering, as mechanical properties should ideally be uniform in all directions to ensure consistent load distribution and structural stability. Additionally, Scaffold #10 (L), despite exhibiting a relatively high compressive stress capacity (96.09 ± 1.72 MPa), suffered from a low pore percentage, which can significantly hinder *nutrient diffusion* and *growth factor transport*, potentially compromising cell viability and tissue regeneration. In contrast, Scaffold #9 (Holes) and Scaffold #1 (Neovius) demonstrated a favorable balance between mechanical stiffness and biological functionality. With stiffness values of 96.58 ± 6.97 MPa and 89.81 ± 3.95 MPa, respectively, both scaffolds exhibited sufficient compression resistance to withstand physiological loading. Furthermore, their adequate porosity and deposition mass make them promising candidates for bone tissue engineering applications, as they allow for enhanced cell infiltration, vascularization, and overall tissue integration compared to the other scaffold designs.

## 4. Conclusions and Future Work

### 4.1. Conclusions

In this study, the mechanical performance of SimuBone material was systematically investigated through tensile and torsion analyses, providing key insights into its strength and deformation behavior under different loading conditions. Additionally, the mechanical properties of 10 TPMS scaffold designs (composed of SimuBone) were evaluated, focusing on their compressive stiffness, porosity, and structural integrity. These analyses offered an understanding of the material’s suitability for bone tissue engineering applications as well as the influence of scaffold architecture on mechanical performance. Below is a summary of the main observations.

- SimuBone exhibited a shear modulus of rigidity of 714.79 ± 11.97 MPa, indicating its resistance to angular deformation under applied stress. The material’s average yield strength was 44 ± 1.31 MPa, highlighting its ability to withstand mechanical loads before permanent deformation occurred. Additionally, necking and fracture were observed at approximately 1.5% and 1.75% strain, respectively, marking the onset of material instability and failure.
- Scaffold fabrication with the horizontal orientation (0°) exhibited the highest tensile modulus at 5404.20 ± 192.30 MPa, followed by the diagonal orientation (45°) at 5224.42 ± 173.77 MPa, and the vertical orientation (90°) at 4911.84 ± 175.18 MPa. This trend highlighted the anisotropic nature of SimuBone, where the horizontal orientation (0°) provided superior stiffness and structural integrity, making it the preferred configuration for load-bearing applications. Furthermore, SimuBone’s tensile stiffness closely aligned with that of cortical and cancellous bone, reinforcing its potential as a viable material for bone tissue engineering applications.
- Scaffolds with large voids exhibited a tendency to collapse inward under compressive loading, indicating a critical need for enhanced wall thickness to improve structural integrity. Among the scaffold designs, Schwarz Primitive (P) demonstrated the lowest compressive stiffness at 25.30 ± 1.38 MPa, making it the weakest design, whereas P.W. Hybrid (Vertical) emerged as the strongest with a compressive stiffness of 111.20 ± 2.28 MPa, highlighting its superior load-bearing capacity. Additionally, an inverse relationship was observed between pore percentage and deposition mass. However, no clear correlation was found between deposition mass and compression modulus, emphasizing the complex interplay of scaffold architecture, porosity, and mechanical performance.
- P.W. Hybrid demonstrated high stiffness in the vertical orientation but moderate stiffness in the horizontal orientation, highlighting its anisotropic mechanical behavior. Although suitable for load-bearing applications in specific directions, this characteristic may limit its versatility in bone tissue engineering.
- Scaffold #10 (L) exhibited a high compressive stress capacity, making it structurally robust under mechanical loading. However, its low pore percentage could hinder nutrient diffusion and cellular infiltration, potentially impacting biological integration and tissue regeneration.
- Scaffold #9 (Holes) and Scaffold #1 (Neovius) achieved a favorable balance between mechanical stiffness and biological functionality, offering both structural integrity and adequate porosity for cell growth and nutrient transport. These scaffolds show strong potential for bone tissue engineering applications, combining mechanical durability with enhanced bioactivity.

### 4.2. Future Work

Future work will involve a comprehensive investigation of all 10 TPMS scaffold designs using finite element analysis (FEA) to simulate their structural response under mechanical loading. This analysis will focus on identifying deformation patterns, stress distribution, and potential failure points, providing deeper insight into weak support regions. The findings will help optimize scaffold architectures, enhancing their mechanical performance and durability for bone tissue engineering applications. 

## Figures and Tables

**Table 1:** The governing equations used for the parametric design of the 10 TPMS bone scaffolds.

Equations	TPMS Design
$3\left(\cos\left(x\right)+\cos\left(y\right)+\cos\left(z\right)\right)+4\left(\cos\left(x\right)\cos\left(y\right)\cos\left(x\right)\right)$	(1)
$-\left(\cos\left(x\right)+\cos\left(y\right)+\cos\left(z\right)\right)$	(2)
$\sin\left(x\right)\cos\left(y\right)+\sin\left(z\right)\cos\left(x\right)+\sin\left(y\right)\cos\left(z\right)$	(3)
$\cos\left(x\right)\cos\left(y\right)\cos\left(z\right)-\sin\left(x\right)\sin\left(y\right)\sin\left(z\right)$	(4)
$2\left(\cos\left(x\right)\cos\left(y\right)+\cos\left(y\right)\cos\left(z\right)+con\left(z\right)\cos\left(x\right)\right)\;-(\cos\left(2x\right)+\cos\left(2y\right)+\cos\left(2z\right))$	(5)
$\cos\left(x+\frac{1+\sqrt{5}}{2y}\right)+\cos\left(x-\frac{1+\sqrt{5}}{2y}\right)+\cos\left(y+\frac{1+\sqrt{5}}{2z}\right)\;+\cos\left(y-\frac{1+\sqrt{5}}{2z}\right)+\cos\left(z+\frac{1+\sqrt{5}}{2x}\right)+\cos\left(z-\frac{1+\sqrt{5}}{2x}\right)$	(6)
$10\left(\cos\left(x\right)\cos\left(y\right)\right)+\cos\left(y\right)\cos\left(z\right)+\cos\left(z\right)\cos\left(x\right)\;-0.01(\cos\left(x\right)\cos\left(y\right)\cos\left(z\right))$	(7)
$\sin\left(x\right)\sin\left(y\right)\sin\left(z\right)+\sin\left(x\right)\cos\left(y\right)\cos\left(z\right)\;+\cos\left(x\right)\sin\left(y\right)\cos\left(z\right)+\cos\left(x\right)\cos\left(y\right)\sin\left(z\right)$	(8)
$\cos\left(x\right)+\cos\left(y\right)+\cos\left(z\right)+4\cos\left(x\right)\cos\left(y\right)\cos\left(z\right)$	(9)
$0.5\sin\left(2x\right)\cos\left(y\right)\sin\left(z\right)+\sin\left(2y\right)\cos\left(z\right)\sin\left(x\right)\;+\sin\left(2z\right)\cos\left(x\right)\sin\left(y\right)-0.5\cos\left(2x\right)\cos\left(2y\right)\;+\cos\left(2y\right)\cos\left(2z\right)+\cos\left(2z\right)\cos\left(2x\right)$	(10)

**Table 2:** The main design parameters, along with their values, set for optimal parametric generation of the 10 TPMS bone scaffolds.

Parameters	Values
Model Dimensions (mm^3^)	6 × 6
Iteration Step Size for Designs (b) through (i) Iteration Step Size for Designs (a) and (j)	7.710 18.710
Merged Toggle	True
IsoValue	−0.269
ArrBox (x,y,z) Count	2
Level	1
WBThickness Distance (mm)	0.15

**Table 3:** The main parameters set for the FDM-fabrication of the 10 TPMS bone scaffold designs, torsion bars, and tensile bars.

**Parameter**	**Type**	**Level [Unit]**
Medical Composite	Material	SimuBone (Medical Grade)
Scaffold Dimensions	Design	15 × 15 × 15 [mm]
Layer Height (Thickness)	Design	200 [µm]
Layer (Line) Width	Design	300 [µm]
Infill Density	Design	100 [%]
Nozzle Size	Machine	400 [µm]
Bed Temperature	Machine	60 [°C]
Print Speed	Machine	10 [mm/s]
Deposition Head Temperature	Machine	240 [°C]
Flow (Feed) Rate	Machine	120 [%]
Build Plate Adhesion Type	Machine	Brim

**Table 4:** The standard dimensions used for the design and fabrication of torsion and tensile bars for characterization of the mechanical properties of the TPMS bone scaffold designs.

**Parameters**	**Torsion Bar**	**Tensile Bar**
Type	SM 1001	ASTM D638−14 (Type IV)
Width narrow section (mm)	6	6
Length narrow section (mm)	76.2	33
Width Overall (mm)	-	19
Length Overall (mm)	143	115
Gage Length (mm)	-	25
Distance Between Grips (mm)	-	65
Radius of Fillets (mm)	-	14
Outer Radius (mm)	-	25
Outer Area (mm^2^)	12	-

## Data Availability

The datasets generated in this study and supporting the findings of this article are available from the corresponding author upon reasonable request.
